# Metamaterial-enhanced near-field readout platform for passive microsensor tags

**DOI:** 10.1038/s41378-022-00356-4

**Published:** 2022-03-02

**Authors:** Ke Wu, Guangwu Duan, Xiaoguang Zhao, Chunxu Chen, Stephan William Anderson, Xin Zhang

**Affiliations:** 1 Department of Mechanical Engineering, Boston University, Boston, MA 02215 USA; 2 Photonics Center, Boston University, Boston, MA 02215 USA; 3 Department of Radiology, Boston University Medical Campus, Boston, MA 02118 USA

**Keywords:** Materials science, Electrical and electronic engineering

## Abstract

Radiofrequency identification (RFID), particularly passive RFID, is extensively employed in industrial applications to track and trace products, assets, and material flows. The ongoing trend toward increasingly miniaturized RFID sensor tags is likely to continue as technology advances, although miniaturization presents a challenge with regard to the communication coverage area. Recently, efforts in applying metamaterials in RFID technology to increase power transfer efficiency through their unique capacity for electromagnetic wave manipulation have been reported. In particular, metamaterials are being increasingly applied in far-field RFID system applications. Here, we report the development of a magnetic metamaterial and local field enhancement package enabling a marked boost in near-field magnetic strength, ultimately yielding a dramatic increase in the power transfer efficiency between reader and tag antennas. The application of the proposed magnetic metamaterial and local field enhancement package to near-field RFID technology, by offering high power transfer efficiency and a larger communication coverage area, yields new opportunities in the rapidly emerging Internet of Things (IoT) era.

## Introduction

As a key enabling component in many RFID technology platforms, RFID microsensor tags are increasingly prevalent throughout our lives with the development of modern electronics and micro/nanofabrication technology. Microsensor tags, owing to their miniaturization, can be dispatched ubiquitously to enable large coverage areas or leveraged in small or otherwise hard-to-reach areas to monitor their surrounding environments. Myriad applications of microsensor tags have been reported, ranging from monitoring air pollution to human health and beyond^1,2^. In addition, and pertinent to the work presented herein, microsensor technology has been applied in the oil and gas industry to gain a better understanding of the variations in the physical and chemical environments of oil reservoirs^3–5^. In this case, microsensors can be injected into oil and gas wellbores to form a subsurface sensor network capable of gathering and recording measurements in harsh subsurface environments to characterize temperature, pressure, resistivity, and hydrocarbon concentration, which is of utility in a number of applications, including flow assurance, wellbore integrity, hydraulic fracture, and reservoir characterization. However, the size limitations of the microsensor tags prohibit the use of large-capacity onboard batteries, leading to relatively short sensor lifetimes. To address this shortcoming, passive sensor tags without onboard batteries, which harvest energy from external RF transmission for charging and subsequent data readout, have been proposed^6^. The inherently low nature of radiative power transfer efficiency, due to its omnidirectional pattern, precludes the use of far-field radiation for sensor charging^7^. Alternatively, nonradiative power transfer using near-field magnetic coupling yields the possibility of charging due to its relatively high magnetic field strengths^8,9^. However, the mutual coupling between the transmitter and receiver in the near-field decays rapidly as a function of distance (∝1/*d*^3^), compromising the wide applicability of this approach^10^.

Addressing the aforementioned general and inherent limitations of wireless power transfer has received ongoing and significant effort devoted to improving power transfer efficiency and extending power transfer distance since the turn of the 20th century^11–14^. Based on the governing equations, the power transfer efficiency depends primarily on the coupling strength (k) and the quality factors (Q) of the coupled antennas (i.e., Efficiency ∝ k × Q_transmitter_ × Q_receiver_)^15^. As a result, a number of resonators with high Q-factors have been reported to improve power transfer efficiency by decreasing ohmic and radiative losses, including helical and spiral wire-wound coils, resonant shielded loops, printed coils, dielectric resonators, and cavity mode resonators^16–20^. However, a drawback of a high Q-factor is the resultant decrease in bandwidth, leading to a decrease in the robustness of a wireless power transfer system under external perturbation. Alternative reported routes toward optimizing power transfer efficiency include multicoil systems, which improve the frequency bandwidth^21^, as well as the use of delay coils to control the power transfer route^22^. Finally, as noted above, enhancement of the coupling coefficient also leads to improved power transfer efficiency. To this end, metamaterials represent a powerful approach to manipulating near-field interactions and enhancing the coupling between two resonators in this regime.

Metamaterials refer to a class of artificially structured materials that exhibit unique responses to incident electromagnetic (EM) waves at deep subwavelength scales. With their unique evanescent wave amplification properties, a variety of EM metamaterials have been reported to manipulate and enhance electromagnetic fields^23–27^. In the case of near-field power transfer systems, the focus herein, the most commonly applied approach is the insertion of a metamaterial slab between the transmitter and receiver antennas to amplify the magnetic flux density and thus increase the coupling coefficient or mutual induction, ultimately improving the power transfer efficiency^28–31^. However, these efforts at applying metamaterials to near-field-based power transfer systems, while clearly verifying the possible role of metamaterials in enhancing power transfer efficiency, have failed to address inter-unit cell interactions and the effects of metamaterial configuration on magnetic field strength or distribution. With respect to the specific application to RFID systems, metamaterials have been reported to improve the read range and serve to confine the read area to mitigate undesired communication beyond the expected read area. However, in contrast to the focus on near-field RFID herein, metamaterials have most commonly been applied to far-field-based RFID systems^32–34^.

Previously, we reported the development of a magnetically coupled communication and charging platform to interrogate and recharge microsensor tags^35,36^. In this system, as the coverage area of the transmitting antenna array increases, the relative field strength decreases with a given input power level. Thus, in some cases, the field strength remains below the threshold power density requisite for the microchips to function. Therefore, we developed a field enhancement system featuring two distinct techniques, presented herein, to passively boost the magnetic field strength without increasing the input power: a metamaterial-enhanced reader antenna and a magnetic local field enhancement package (M-LFEP) for the integrated tags. Importantly, these approaches can be applied independently or synergistically as a function of intended application to yield magnetic field enhancement. Our system is capable of interrogating microsensor tags featuring ultrasmall antennas with areas of 0.38 × 0.38 mm across a functional distance for diverse applications, including IoT and health care uses, among others. To the best of our knowledge, the tag antenna employed in our readout platform and reported herein represents the smallest near-field UHF antenna. In this article, the proposed readout platform is analytically and numerically investigated and then experimentally verified through near-field tag-reading measurements, demonstrating the feasibility and effectiveness of magnetic metamaterials in enhancing near-field power transfer efficiency.

## Results and discussion

### System overview

**Fig. 1:**
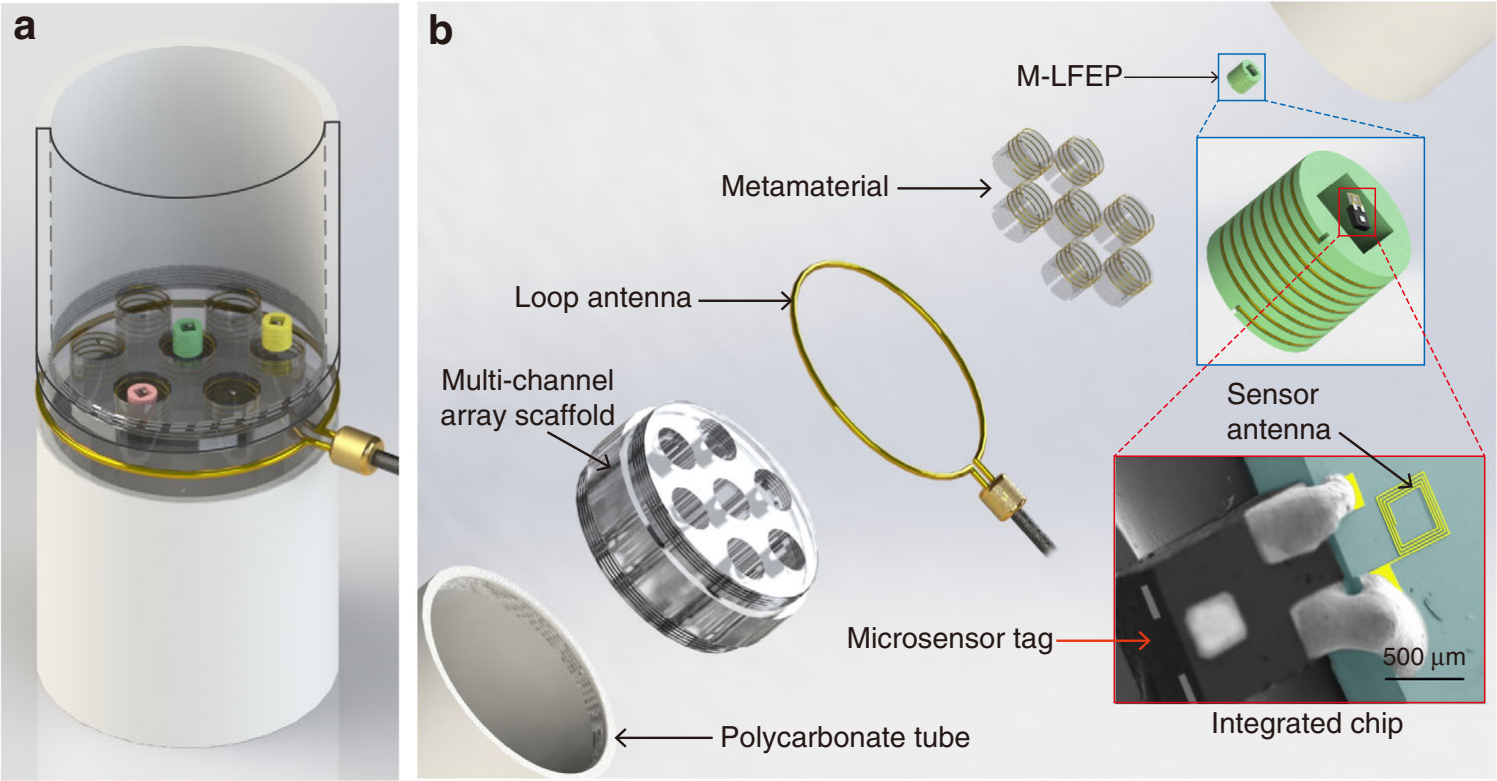
Metamaterial-enhanced near-field readout platform for passive microsensor tags. **a** Schematic view of the readout platform. **b** Exploded view of the readout platform featuring magnetic metamaterial and magnetic local field enhancement package (M-LFEP)-enabled field enhancement approaches. The inset shows a false-color SEM image of a fabricated integrated chip featuring a microsensor tag and readout antenna.

The proposed near-field, ultrahigh frequency (840–960 MHz) RFID readout platform for data interrogation from passive microsensor tags is shown in Fig. 1, which is composed of a fluidic channel with embedded ‘multichannel’ array scaffolding, in which the metamaterials are located. The reader antenna is a loop antenna enhanced by magnetic metamaterials composed of an array of unit cells featuring metallic helices. When excited by an external oscillator circuit, the reader antenna transmits an interrogating signal and demodulates a backscattered signal from the tags. On the microsensor tag side, the green solenoid in Fig. 1 represents the proposed M-LFEP operating at the same resonance frequency as the microsensor tags. The coupling between the reader antenna and the M-LFEP on the tags is achieved with a resonance match. The strong magnetic induction in the M-LFEP serves to enhance the local magnetic field, and when in proximity to the reader antenna, the tag antenna resonantly harvests energy from the reader antenna to activate and transmit its stored data. The relatively large size of the metamaterial when compared to the sensor antenna is designed to expand the reading area of the readout platform. The large reading area allows this reader to simultaneously process multiple tags within its antenna field, thereby enabling an automated and high-throughput readout platform for interrogating massive numbers of miniaturized tags in a short period of time.

### Metamaterial design and analysis

**Fig. 2:**
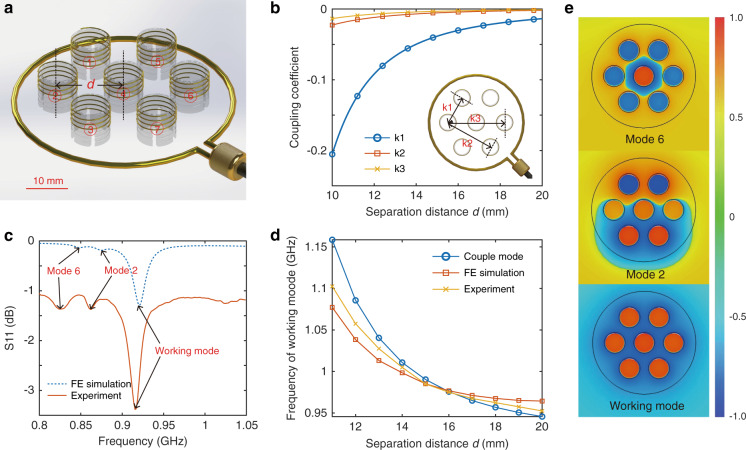
Metamaterial properties. **a** Metamaterial composed of a unit cell array featuring metallic helices (serial numbers of unit cells are labeled for the subsequent analyses). **b** Coupling coefficients as a function of unit cell separation distance *d* derived from theoretical analyses. **c** Simulated and experimental reflection coefficient of the metamaterial demonstrating different resonant modes with distinct dips. **d** Frequency of the working mode of the metamaterial as a function of separation distance *d* of unit cells. **e** Magnetic field distribution at the three resonance frequency points: 845, 870, and 915 MHz. The magnetic field strength is normalized to its maximum value.

The magnetic metamaterial reported herein is comprised of an array of metallic helices distributed in a hexagonal configuration (Fig. 2a), composed of an electrically conducting wire of total length *l*_*t*_ and cross-sectional radius *a*_*mm*_ wound into a helix of *n*_*mm*_ turns, radius *r*_*mm*_, and height *h*_*mm*_. The coupling of individual unit cells in the metamaterial array gives rise to a synergy and bulk effective material property. The unit cell synergy yields a resonant mode in which the direction of the electric current induced by an external magnetic field is identical in each unit cell. These currents, upon induction by an external magnetic field, lead to a dramatic enhancement of the local magnetic field. With respect to RFID, when the frequency of the resonant mode approximates the working frequency of the RFID system, the enhancement of the local magnetic field leads to a dramatic increase in the mutual induction between resonators, thereby improving the power transfer efficiency between the reader and tag antennas.

In an effort to elucidate the factors influencing the resonant mode of the metamaterial, electromagnetic theory is initially employed to define the equivalent self-inductance and mutual inductance of discrete unit cells as^15^:

1 $$L_{ij} = \frac{{\mu _0}}{{4\pi \left| {I_iI_j} \right|}}{\int\!\!\!\!\!\int} {dr_idr_j} \frac{{J\left( {r_i} \right)J\left( {r_j} \right)}}{{\left| {r_i - r_j} \right|}}$$

where *r*_*i*_ and *r*_*j*_ are the integration elements along the path of the helix, *I*_*i*_ and *I*_*j*_ are the equivalent electric currents in the helix, and *J(r*_*i*_*)* and *J(r*_*j*_*)* are the current densities (vectors) at *r*_*i*_ and *r*_*j*_. When *i* is equal to *j*, Eq. (1) calculates the self-inductance; when *i* and *j* differ, Eq. (1) yields the mutual inductance. In the resonant mode, the amplitude of the electric current follows a sinusoidal profile along the helical wire, with a maximum value (*I*_*0*_) at the center and zero at the ends of the wire; thus, $L_{i,j} = I_0/\sqrt 2$. Both the mutual capacitance and the self-capacitance may be derived via the inverse of the coefficients of the potential matrix and expressed as^37^:

2 $$P_{ij} = \frac{1}{{4\varepsilon _0\varepsilon _{{{\mathrm{r}}}}\left| {Q_iQ_j} \right|}}{\int\!\!\!\!\!\int} {dr_idr_j} \frac{{\rho \left( {r_i} \right)\rho \left( {r_j} \right)}}{{\left| {r_i - r_j} \right|}}$$

3 $${{{\mathrm{C}}}} = \left[ {\begin{array}{*{20}{c}} {C_{11}} & \cdots & {C_{1m}} \\ \vdots & \ddots & \vdots \\ {C_{m1}} & \cdots & {C_{mm}} \end{array}} \right] = \left[ {\begin{array}{*{20}{c}} {P_{11}} & \cdots & {P_{1m}} \\ \vdots & \ddots & \vdots \\ {P_{m1}} & \cdots & {P_{mm}} \end{array}} \right]^{ - 1}$$

in which *Q*_*i*_ and *Q*_*j*_ are the equivalent charge amounts in the helix and *ρ(r*_*i*_*)* and *ρ(r*_*j*_*)* are the charge densities (scalars) at *r*_*i*_ and *r*_*j*_. As the phase difference between the electric current and charge distribution is *π/2*, the charge density is at a maximum at the ends of the wire *(q*_*0*_*)* and zero at its mid-portion. Considering the charge distribution, the charge amounts in the helix could be expressed by $Q_{i,j} = q_0l_t\pi /\sqrt 2$. The self-capacitance is determined by the diagonal elements of the capacitance matrix **C**, while the remaining elements yield the corresponding mutual capacitance.

Once arranged in a metamaterial array, the effect of coupling between discrete unit cells must be considered, given its effect on the resonant mode of the array. Given their identical geometry, the coupling coefficient of adjacent helical unit cells may be expressed as *k* = *C*_*m*_/*C* + *L*_*m*_/*L*, in which *C*_*m*_ and *L*_*m*_ represent the mutual capacitance and mutual inductance, respectively, and *C* and *L* represent the self-capacitance and self-inductance, respectively. There are three distinct coupling coefficients, namely, *k1*, *k2* and *k3*, in the hexagonally distributed unit cells due to different relative distances, as shown in the inset of Fig. 2b. The total coupling coefficients (*k1*, *k2* and *k3*), including the contributions from capacitance coupling and inductance coupling, are plotted as a function of the unit cell separation distance *d* of the metamaterial array in Fig. 2b.

Given the interunit cell coupling, the resonant mode of the metamaterial may be derived by employing coupled mode theory and solving the following equation system^38^:

4 $$\frac{{da_n\left( t \right)}}{{dt}} = - \left( {j\omega _0 + \Gamma _n} \right)a_n\left( t \right) + j\mathop {\sum }\limits_{k = 1}^{m,k \ne n} {\rm K}_{kn}a_k\left( t \right),\,n = 1, \cdots ,m$$

in which $\omega _0 = 1/\sqrt {LC}$ represents the resonant angular frequency of a single helix, $\Gamma _n$ represents the decay rate introduced by the material and radiation losses, *K*_*kn*_ is the coupling factor between unit cells and is related to the coupling coefficient *k* as *K*_*kn*_ = *kω*_0_/2, and *m* is the total number of unit cells. Omitting the decay rates of each unit cell for simplicity, only the coupling between adjacent unit cells is considered in deriving the resonant modes of the metamaterial. The resonant mode and the resonant strength of each unit cell may be derived by utilizing the coupling coefficients and coupled mode theory, as shown in Eq. (4). For a hexagonally configured array with a separation distance of 14 mm, the eigenvalue of the resultant matrix is:

5 $$A = - \left[ {916,\,851,\,851,\,813,\,813,\,803,\,798} \right] \times 10^6$$

Each eigenvalue corresponds to the frequency of a specific resonant mode. By solving the eigenvector of the resultant matrix, we derive the resonant strength of each unit cell in the metamaterial, the results of which are listed in Table 1. Each column in the table corresponds to the resonant strength of the individual unit cells at specific resonant modes with the resonant frequency derived from the eigenvalues of the resultant matrix (see unit cell numbering in Fig. 2a). The negative sign, when present, denotes the fact that the electric current is in the opposite direction. It is clear that the direction of the electric current is identical in each unit cell in the case of the resonant mode with the highest resonant frequency, referred to as the working mode for magnetic field enhancement. To verify these theoretical results, finite element (FE) simulation frequency domain (FD) testing experiments were also conducted to derive the reflection coefficient of the metamaterial. Similar to the analytical results above, distinct resonant modes were also identified as discrete dips on the plotted curves derived from FE simulations and FD testing experiments (Fig. 2c); the simulated magnetic field distributions at the three resonance frequencies of 845, 870, and 915 MHz are shown in Fig. 2e. The colors correspond to the resonant strength of each unit cell derived from the simulation results. At a frequency of 845 MHz, the helix in the middle resonates with a magnetic field direction opposing that of the outer helices. At the higher resonant frequency (870 MHz), the magnetic field demonstrates an antisymmetric pattern with a very small magnetic field in the three helices in the middle, while at the highest resonant frequency (915 MHz), the magnetic field distributes uniformly throughout all six peripheral helices, with a slightly stronger field strength in the middle helix. However, unlike the analytical results from which 7 distinct resonance modes were originally derived (Table 1), we only observed 3 distinct resonance frequencies using simulations/experiments, which may be due to the merging of small resonant peaks with nearby resonance modes. Finally, the frequency of the metamaterial’s working mode as a function of unit cell separation distance *d* was calculated based on coupled mode theory (CMT) by solving Eqs. (1)–(4), compared with the simulation and experimental results, and illustrated in Fig. 2d. Importantly, the tunability resulting from variations in separation distance yields powerful design flexibility for this metamaterial. The differences present in Fig. 2d between the simulation/experimental results and the coupled mode theory may be due to the modeling of the scaffold, which was modeled as a uniformly distributed material surrounding the helix array with an averaged permittivity in the analytical calculations, an approximation that differed from subsequent simulations/experiments.

**Table 1:** Resonant strength of the unit cells in the metamaterial at specific resonant modes

Resonance strength	Working mode (916 MHz)	Mode 2 (851 MHz)	Mode 3 (851 MHz)	Mode 4 (813 MHz)	Mode 5 (813 MHz)	Mode 6 (803 MHz)	Mode 7 (798 MHz)
Unit cell #1	−0.35	0.085	0.57	0.16	−0.56	−0.22	0.41
Unit cell #2	−0.35	0.54	0.21	−0.56	0.14	−0.22	−0.41
Unit cell #3	−0.35	0.45	−0.36	0.40	0.41	−0.22	0.41
Unit cell #4	−0.53	1.81 × 10^−15^	4.35 × 10^−16^	−5.38 × 10^−15^	−7.64 × 10^−15^	0.85	1.24 × 10^−14^
Unit cell #5	−0.35	−0.085	−0.57	0.16	−0.56	−0.22	−0.41
Unit cell #6	−0.35	−0.54	−0.21	−0.56	0.14	−0.22	0.41
Unit cell #7	−0.35	−0.45	0.36	0.40	0.41	−0.22	−0.41

### Magnetic field distribution and field enhancement

**Fig. 3:**
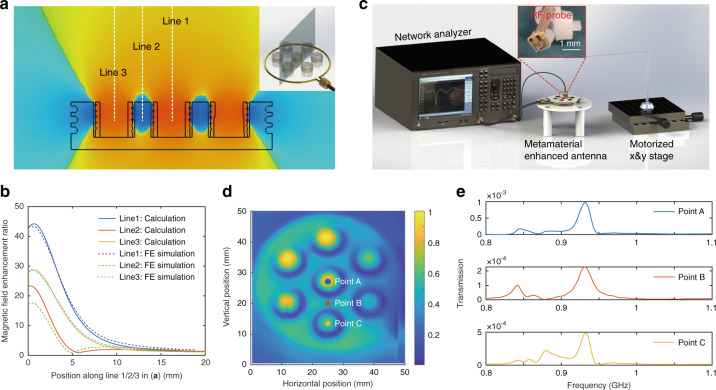
Magnetic field enhancement ratio of the metamaterials. **a** Magnetic field strength along the axial direction distributed along the metamaterial cross-section (depicted as the gray plane in the inset). **b** Magnetic field enhancement ratio as a function of distance from the center of the metamaterial array (along the white dashed line in **a**) with simulation and analytical calculation results in solid and dashed lines. **c** Conceptual drawing of the experimental setup for near-field magnetic mapping. Inset shows a photo of microprobe. **d** Experimental magnetic field map on a parallel plane 2 mm above the metamaterial surface. **e** Spectra of the magnetic field distribution at points A, B and C, as shown in **d**.

After matching the working mode frequency of the metamaterial to the working frequency of the RFID system, the working mode may be excited by the magnetic field *B*_*0*_ generated by the feeding loop. The magnetic field *B*_*0*_ induces an AC voltage across the helical unit cell as *V* *=* *n*_*mm*_*SB*_*0*_*/dt*. The impedance of the helix may be expressed as *Z*_i_ = j*ωL* + 1/j*ωC* + R, in which *L* and *C* represent the equivalent inductance and capacitance of the unit cells in the array, respectively, and *R* represents the equivalent resistance, composed of the ohmic loss of the wire, the radiation loss of the helices, and the dielectric loss of the scaffolding material. Thus, the electric current within a metallic helix may be calculated as *I* = *V/Z*_i_. The induced magnetic field contains two components, one in the axial direction and the second in the radial direction of the helix. To simplify the analysis, the orientation of the tag antenna is assumed to be aligned in the axial direction of the loop antenna and the metamaterial unit cells. As such, only the magnetic field component along the axial direction of the helix contributes to the magnetic field enhancement and can be calculated as:^39^

6 $${{{\mathrm{B}}}}_z = \frac{{\mu _0n_{mm}I_0}}{{4\pi h_{mm}}}\int_{ - \frac{h}{2}}^{\frac{h}{2}} \cos \left( {\frac{{\pi z^{\prime} }}{{h_{mm}}}} \right)\int_0^{2\pi } \frac{{r_{mm} - \rho \cos \left( {\varphi^{\prime} } \right)}}{{\left[ {\rho ^2 - 2r_{mm}\rho \cos \left( {\varphi^{\prime} } \right) + {r_{mm}}^2 + \left( {z - z^{\prime}} \right)^2} \right]^{\frac{3}{2}}}}d\varphi^{\prime} dz^{\prime}$$

in which *n*_*mm*_, *r*_*mm*_, and *h*_*mm*_ are the number of turns, radius, and height of the helical coil in the metamaterial, respectively. *I*_*0*_ represents the amplitude of the electric current in the mid-portion of the helix, *ρ* and *z* represent the polar coordinates of the target point, cos*(*π*z*^*’*^*/h*_*mm*_*)* reflects the current distribution along the helix, and the polar coordinates are depicted in Supplementary Fig. S1 in the Supplementary Materials. With the strength of the electric current in each unit cell designated by the eigenvectors of the coupled mode theory, the magnetic field enhancement ratio may be calculated by summing the magnetic field components (including both B_0_ and the magnetic fields generated by each unit cell) and normalizing to the initial field B_0_. Figure 3a depicts the simulated magnetic field distribution at the center of the metamaterial array along the axial direction (analysis location depicted in the highlighted cross-section of the inset). In addition, the field enhancement ratio along the white dashed line, as a function of distance from the metamaterial array, is also plotted in Fig. 3b, demonstrating a high degree of agreement with the theoretical results.

To experimentally demonstrate the validity of the theoretical analyses, we developed a near-field measurement setup to map the magnetic field distribution surrounding the metamaterial. Figure 3c illustrates the schematic of the setup utilized in this work, featuring a loop antenna that was connected to port 1 of the network analyzer through a 50-ohm coaxial cable. The RF signal fed into the loop antenna served to excite the inner metamaterial. The tip of the microprobe consisted of a spiral antenna and a coupling loop with a 500 × 500 µm area, as shown in the inset of Fig. 3c, which was connected to port 2 of the network analyzer via a coaxial cable. The miniaturized size of the probe served to reduce its impact on the field distribution of the metamaterial, ensuring a high spatial resolution of the magnetic field mapping. The coaxial cable was attached to a thin acrylic rod, which was mounted to a two-dimensional adjustable stage incorporating a computer-controlled motor drive. The transmission coefficient, i.e., S21, was used as an indicator of the magnetic field strength. By adjusting the relative position between the metamaterial and probe, the magnetic field surrounding the metamaterial was mapped. To ensure high accuracy of this measurement, we matched the resonance frequency of ~ 932 MHz between the metamaterial and probe, which vastly improved the coupling between the two. The normalized magnetic field strength on a parallel plane 2 mm above the metamaterial is depicted in Fig. 3d. It is evident that the magnetic field peaks at the center of each helical resonator unit cell in the metamaterial. The spectra of the magnetic field distribution are shown in Fig. 3e at distinct locations labeled points A, B and C in Fig. 3d. For the unit cells, the magnetic field is maximal at a resonance frequency of 932 MHz.

### Magnetic local field enhancement package (M-LFEP)

**Fig. 4:**
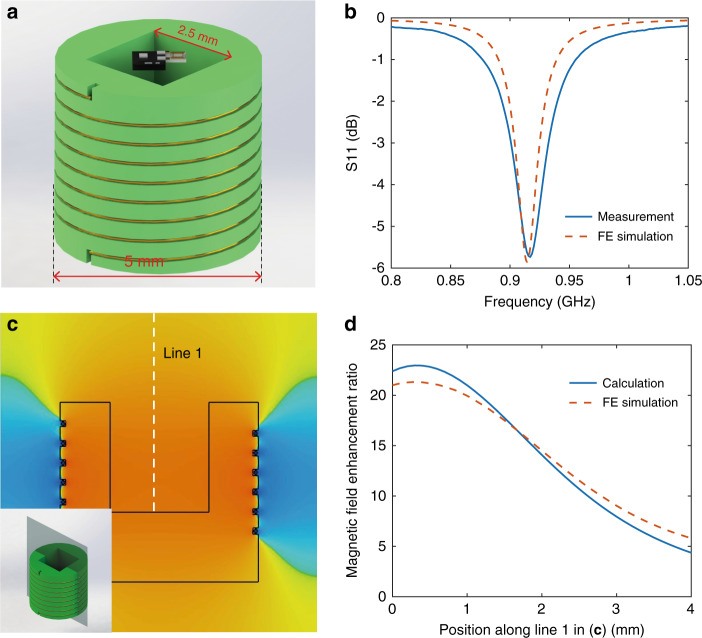
Properties of M-LFEP. **a** Schematic of the M-LFEP embedded with an integrated tag. The side length of the cavity is 2.5 mm larger than the size of the microchip (<1 mm). Inset: photographs of the fabricated M-LFEP and magnified silver bonding wire. **b** Experimental and simulated resonance frequency of the M-LFEP. **c** Magnetic field distribution in the resonant state along the axial direction at the cross-section depicted as the gray plane in the inset. **d** Magnetic field enhancement ratio as a function of position along the white dashed line in **c.**

In addition to the metamaterial-enhanced reader antenna, we also developed the M-LFEP component to passively boost the magnetic field strength without increasing the input power. The M-LFEP includes a helical coil resonator wound around a package cavity, as shown in Fig. 4a. When the coil is activated by an external alternating magnetic field at its resonant frequency, a strong current is excited inside the resonator, generating a high-strength magnetic field with the frequency of the activating field. Given the magnetic field distribution of the helical coil, which is highly concentrated inside the coil, the power transfer efficiency is dramatically increased for the purposes of wireless charging and communication by positioning the microchip within the M-LFEP cavity; this concept is illustrated in Fig. 4a. To maximize the coupling between the reader antenna and the M-LFEP, matching the resonance frequency of the M-LFEP to the working frequency of the microsensor tags is required. By precisely designing the diameter of the helix, the number of turns, and the spacing between each turn, the resonance of the helical resonator was set at approximately 915 MHz, as shown in Fig. 4b. Similar to the previous analyses of the metamaterial, we studied the magnetic field strength within the M-LFEP as well as its field enhancement ratio. The effective inductance and capacitance of the M-LFEP may be extracted using Eqs. (1)–(3). The magnetic field strength centrally within the M-LFEP was calculated to have been improved by 23-fold. To validate the analytical results, a simulation for the M-LFEP was also performed. Figure 4c depicts the magnetic field strength distribution along the axial direction at the cross-section depicted as the gray plane in the inset. From this field distribution pattern, the maximum magnetic field is found to occur in the center of the M-LFEP, the location at which the microsensor tag would be placed. Figure 4d demonstrates the magnetic field enhancement ratio as a function of distance from the bottom to the top of the cavity (along the white dashed line), demonstrating a high degree of agreement between the theoretical and simulation results.

### Metamaterial and M-LFEP combined performance

**Fig. 5:**
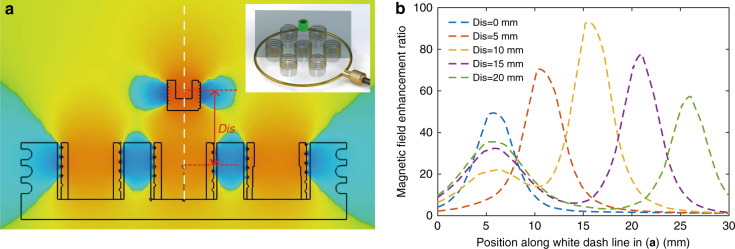
Magnetic field distribution of the combined metamaterial and M-LEFP. **a** Magnetic field strength along the axial direction distributed along the metamaterial and M-LFEP cross-section (depicted as the gray plane in the inset). **b** Magnetic field enhancement ratio as a function of distance from the center of the metamaterial array (along the white dashed line in **a**) from simulation results.

From the above analyses, we learned that a metamaterial-integrated loop antenna serves to enhance the magnetic field strength on the reader antenna side. Similarly, on the microsensor tag side, the M-LFEP approach may be employed to dramatically improve the local magnetic field strength. In addition, the field enhancement ratios *ν1* by the metamaterial and *ν2* by the M-LFEP were derived based on coupled mode theory and the Biot–Savart law and subsequently verified by FE simulation. Finally, the metamaterial and M-LFEP component can be used synergistically in combination to enhance the magnetic field strength. However, the combined field enhancement ratio is not a simple multiplicative or additive relationship between *ν1* and *ν2* due to varied interactions between these two components as a function of their separation distance. To investigate the combined performance of these two components, we simulated the magnetic field distribution for the combination of the metamaterial and M-LFEP. The simulated magnetic field distribution at the center of the metamaterial array and the M-LFEP along the axial direction (analysis location depicted in the highlighted cross-section of the inset) are depicted in Fig. 5a. In addition, the field enhancement ratio along the white dashed line at different distances *Dis* between the metamaterial array and M-LFEP are plotted in Fig. 5b. From the simulation results, we demonstrate that the synergy between the metamaterial and M-LFEP yields a higher degree of improvement in magnetic field strength when compared with the results of the metamaterial or M-LFEP acting alone. From the plot in Fig. 5b, it is evident that there is a critical distance between these two components, at which the field enhancement ratio may be boosted to its maximal value. In terms of this specific setup, when the separation distance of these two components is ~15.5 mm, the magnetic field enhancement ratio in the center of the M-LFEP reaches its maximal value of 93. However, the field enhancement ratio decreases when the metamaterial and M-LFEP move to shorter or longer separation distances. In the case of shorter distances, the strong mutual inductance between the metamaterial and M-LFEP weakens the electric current in unit cell #4 (see unit cell numbering in Fig. 2a). As a result, the magnetic field arising from the induced current is also reduced. In the case of longer distances, the interaction between these two components becomes weaker, and the induced current in the M-LFEP results primarily from the initial magnetic field *B*_*0*_. Therefore, the field enhancement ratio, without any significant contribution from the metamaterial, is relatively low when compared with the optimal distance. In conclusion, under specific and optimized conditions, when demanding maximally high magnetic field strength or power transfer efficiency, the metamaterial and M-LFEP may readily be employed in combination to boost the magnetic field strength.

### Power transfer efficiency

As previously stated, the metamaterial and M-LFEP can be used to boost the magnetic field passively without an increase in input power. However, the enhancement in magnetic field strength is not linearly related to the improvement in power transfer efficiency for the RFID system. Even though the power received by the sensors is improved due to the field enhancement, the introduction of the metamaterial and local resonator also consumes energy, including ohmic loss from the conducting wire, dielectric loss from the scaffolding material, and radiation dissipation to the surrounding space. Therefore, it is necessary to evaluate the enhancement of the power transfer efficiency for the RFID system when the metamaterial or M-LFEP is applied. To perform this evaluation, we designed and fabricated microantennas, which were combined with a commercially available passive RFID tag to form an integrated tag with a working frequency range from 840 to 960 MHz, as shown in the bottom right of Fig. 1. By optimizing the dimensions of the tag antenna, the microantenna inductive can be used to cancel the capacitance of the RFID tag to ensure an impedance match. Further details about the optimization process and the fabrication results for the integrated tag are presented in the Supplementary Materials.

**Fig. 6:**
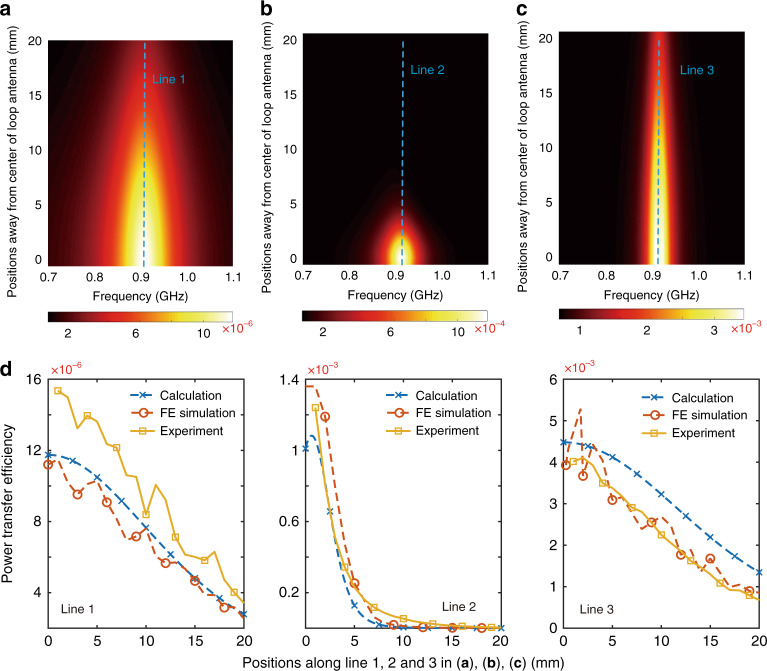
Evaluations on power transfer efficiency. **a** Power transfer efficiency without enhancement as a function of the working frequency and distance from the center of the loop antenna. **b** Power transfer efficiency enhanced by the metamaterial. **c** Power transfer efficiency enhanced by M-LFEP. **d** Power transfer efficiency as a function of distance from the center of the loop antenna at a fixed working frequency of 915 MHz (along blue dashed lines in **a**, **b**, and **c**). *Note different power transfer efficiency scales in **a**–**d**.

Herein, we employed three experimental setups of the RFID system: Setup 1: Loop antenna + integrated tag, the system without enhancement as a reference. Setup 2: Metamaterial-enhanced loop antenna + integrated tag, the system enhanced by the metamaterial. Setup 3: Loop antenna + M-LFEP-enhanced integrated tag, the system enhanced by the M-LFEP. To evaluate the power transfer efficiency, the power dissipation in all components was calculated (please see Supplementary Materials for further details). The calculation results of the power transfer efficiency for setups 1, 2 and 3 are depicted in Fig. 6a–c, respectively. The power transfer efficiency is a function of the working frequency and distance from the center of the loop antenna. Figure 6d depicts the power transfer efficiency along the blue dashed lines shown in Fig. 6a–c, presenting the efficiency for different setups as a function of distance from the center of the loop antenna at a fixed working frequency of 915 MHz. Furthermore, to validate these calculation and simulation results, experiments were also performed to test the power transfer efficiency, and the experimental results are plotted in Fig. 6d. By comparing the calculation, simulation and experimental results for the power transfer efficiency, a high degree of agreement between these results can be observed, indicating the validity of the theoretical model. Based on the datasheet of the RFID tag, the minimum operating power supply for the tag is −15 dBm. Since the transmitting power of the reader circuit is 20 dBm, the power transfer efficiency between the transmitter and receiver must be at least 3.16 × 10^−4^ to ensure communication. Figure 6d indicates that setup 1, in which the reader antenna was composed of a loop antenna with a diameter of 50 mm, did not enable functional communication between the reader and sensor due to its low efficiency. For setup 2, in which the loop antenna was enhanced by the metamaterial, the power transfer efficiency was improved to 1.08 × 10^−3^ at the center of the central unit cell of the metamaterial. At the center position of the peripheral unit cells, the power transfer efficiency was boosted to 4.57 × 10^−4^, which was sufficient to power up the RFID tag. In comparison to prior works with near-field ultrahigh frequency (UHF) RFID, our system is capable of interrogating miniaturized sensor tags that feature the smallest antennas reported to date, with an area of 380 × 380 μm (please see Supplementary Materials for further details). For setup 3, the power transfer efficiency is improved to 3.16 × 10^−3^ with the use of the M-LFEP when the M-LFEP is located in the center of the loop antenna. When the M-LFEP position deviated from the center of the loop antenna, the power transfer efficiency decreased. Nevertheless, owing to the field enhancement by the M-LFEP, the communication distance between the reader antenna and the RFID tag could be extended up to 32.8 mm, the point at which the power transfer efficiency decreased below the minimum level required to power up the integrated tag. Based on the analytical results, the following series of conclusions can be drawn. First, with a perfect resonance match, the power transfer efficiency can be improved by several hundred-fold using the enhancement techniques reported herein. In addition, the M-LFEP method affords improved performance when increasing the distance between the reader antenna and sensor chip. This analysis and the relative merits of power transfer enhancement using metamaterial-enabled or local resonator approaches can offer insight into system design for myriad future practical application scenarios. It should be noted that the efficiency could be further improved by matching the geometric size of the metamaterial and sensor antennas. However, considering practical application scenarios, the goal of our work lies not only in acquiring an ultimate high efficiency but also in achieving a large readout platform reading area. On the basis of ensuring the intended functionality of the readout platform, we designed the metamaterial to make the reading area as large as possible. The large reading area allows this reader to simultaneously process multiple tags within its antenna field. On the premise of ensuring the sensor antenna size at the micro level, the balance between a large reading area and power transfer efficiency enables an automated and high-throughput platform for miniaturized sensors.

### Experimental RFID validation

**Fig. 7:**
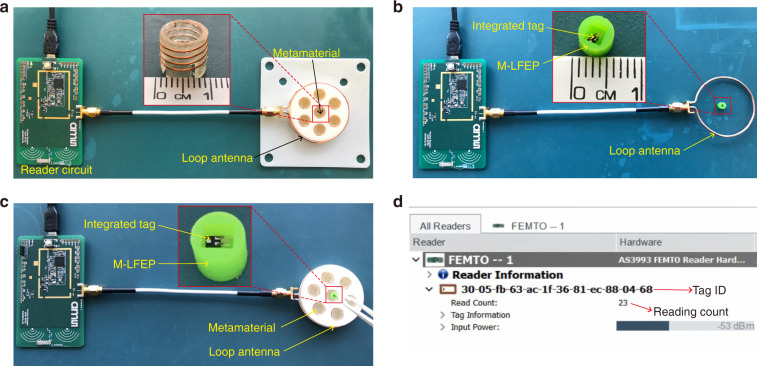
Experimental validation. **a** Overview of the experimental setup for testing the metamaterial-enhanced reader antenna. The inset in the middle demonstrates a single unit cell of the metamaterial. **b** Overview of the experimental setup for testing the M-LFEP. The inset in the middle demonstrates the M-LFEP and the embedded integrated tag. **c** Overview of the experimental setup for testing the combination of the metamaterial and M-LFEP. The inset in the middle demonstrates the M-LFEP and the embedded integrated tag. **d** The screenshot of the graphical user interface displaying the readout information from the integrated tag.

To demonstrate the capacity for wireless communication with the enhancement from the metamaterial-enhanced reader and the M-LFEP, the experimental setup shown in Fig. 7 was employed. The detailed module composition in the RFID system and the readout process are explained in the Supplementary Materials. In the setup, an off-the-shelf RFID integrated chip (IC) bonded with a miniaturized antenna was used as a communication module for the integrated tag, as shown in the inset of Fig. 7a. The reader antenna was connected to a commercially available open-source RFID reader circuit, which was compatible with the aforementioned chip. In setup 1 (Fig. 7a), the loop antenna was enhanced by the metamaterial. In setup 2 (Fig. 7b), a loop antenna was employed as the reader antenna, connected to the Femto Reader via a 50-ohm coaxial cable. On the integrated chip side, we 3D printed a package with grooves defining a helical shape along its outer surface, with silver wire subsequently wound into the grooves to form the M-LFEP. The integrated tag was placed in the center of the M-LFEP to achieve enhanced communication, as shown in the inset figure in the middle in Fig. 7b. In setup 3 (Fig. 7c), both the metamaterial and M-LFEP were employed in the readout platform to boost the wireless power transfer and communication efficiency. When the integrated tag was aligned with the reader antenna, it was charged and transmitted its ID number to the reader based on the UHF EPC global Gen 2 standard. Once communication was established, the interrogated information from the RFID tag was shown by the AS3993 Reader Suite, a program displaying the tag ID and reading count in real-time, as shown in Fig. 7d. As long as the antennas remained coupled, the reader continued to interrogate the tags and count the number of interrogations. As a reference, when a loop antenna was employed as the reader antenna without enhancement from either the metamaterial or the M-LFEP, the Femto Reader failed to interrogate the ID number and commands stored in the RFID tag. Based on a comparison of the experimental results (see videos 1, 2 and 3 in the Supplemental Materials), the readout platform enhanced by the metamaterial or M-LFEP enabled successful communication when the RFID tag passed through the center of the loop antenna, demonstrating the effectiveness of these two distinct magnetic field enhancement techniques. When the metamaterial and M-LFEP were employed simultaneously, the communication distance between the loop antenna and sensor antenna could be further improved, which agrees well with the previous simulation results of magnetic field enhancement.

## Materials and methods

### Geometry of the metamaterial and M-LFEP structure

The geometries of each helix in the metamaterial array, as shown in Fig. 2a, were 4.69 mm, 4.5 mm, 0.125 mm, and 3.85, representing unit cell height, radius, wire diameter and number of turns, respectively. The wires of the helices were composed of copper, and the permittivity of the scaffolding core material was assumed to be (3.5 + 0.04j). The M-LFEP, as shown in Fig. 4a, featured geometries of 3.5 mm, 2.5 mm, 0.01 mm, and 7 for its height, radius, wire diameter and number of turns, respectively. The wire of the helical coil of the M-LFEP was composed of silver, and the permittivity of the scaffolding core material was assumed to be (4 + 0.16j).

### Fabrication and geometry of the microsensor tag antenna

The microsensor tag antenna was fabricated by electroplating copper on a 500-µm thick Pyrex glass substrate. The sensor antenna featured a double-layer spiral structure separated and supported by a layer of SU8. The two spiral layers of copper were interconnected in the middle. The geometries of the double-layer spiral antenna, as shown in Fig. 1, were 10 μm, 5 μm, 10 μm, and 380 μm for wire width, wire thickness, space between neighboring turns, and outer side length of the spiral square coil.

### Near-field mapping for metamaterial

The microprobe employed in the experimental setup consisted of a spiral antenna (same as the tag antenna) and a coupling loop with a 500 × 500 µm area. The transmission coefficient between the metamaterial and microprobe was measured using a vector network analyzer (VNA, E5071C Keysight Inc.). In addition, the resonance frequencies of the antennas, metamaterial, and M-LFEP were tuned and measured with this VNA by utilizing a coupling loop.

### Experimental setup for RFID validation

In the RFID validation experiments, a UHF RFID single-chip reader (AS3993, ams AG Inc.) was used as the reader circuit, supporting the EPC global Class 1 Generation 2 UHF RFID standard. An off-the-shelf RFID IC chip (SL3ICS1002/1202, NXP semiconductors Inc.) integrated with a miniaturized antenna was used as a communication module for the integrated tag, as shown in the inset of Fig. 1, which was compatible with the AS3993 reader circuit.

## Conclusion

By using a magnetic metamaterial, an M-LFEP, or their combination, we developed an efficient UHF RFID readout platform for the interrogation of passive microsensor tags, which yields tremendous opportunities for near-field-based RFID technology in diverse applications. Moreover, with the dramatically increased use of autonomous electronic devices (laptops, cell phones, electric cars, robots, implantable devices, etc.), near-field enhancement techniques may open up new possibilities for near-field power transfer-based IoT applications, such as wireless charging and near-field communication, among numerous other uses. To make these techniques more feasible and convenient in practical applications, future efforts can be directed toward realizing tunability of the metamaterial, thereby ensuring a perfect resonance match, since a minor shift of resonance frequency translates to a significant negative impact on the transfer efficiency. In addition, since the misalignment between the local resonator and reader antenna markedly decreases efficiency, a triaxial local resonator can be developed to achieve omnidirectional field enhancement. Finally, the development of flexible, sheet-like metamaterials represents a promising direction to support increasingly seamless integration of this technology in a variety of conditions; for example, reader antennas may be directly attached to the outer surface of a tube/pipe without interfering with the central fluid. In summary, we report the development of a metamaterial and resonator-enhanced radio frequency readout platform for passive microsensors, which dramatically increases the coverage area and bandwidth of the communication between the sensor chip and reader circuits. Such an enhancement technique would greatly expand the application scope of near-field-based RFID systems in the field of wireless communication and inductive charging.

## Supplementary information

Supplementary information_text

Supplementary video 1

Supplementary video 2

Supplementary video 3

## Data Availability

All data needed to support the conclusions in the paper are presented in the paper and the Supplementary Information. Additional data related to this paper may be requested from the authors.
