# Supplementary material for: Metamaterial-enhanced near-field readout platform for passive microsensor tags
Source: Microsyst Nanoeng. 2022 Mar 2;8:28. doi: 10.1038/s41378-022-00356-4 (PMC8891326; doi:10.1038/s41378-022-00356-4)
Supplement: Supplementary file 1 — Supplementary information_text [file 41378_2022_356_MOESM1_ESM.docx]

Supplementary Information for

**Metamaterial-enhanced near-field readout platform for passive microsensor tags**

Ke Wu^1,2#^, Guangwu Duan^1,2#^, Xiaoguang Zhao^1,2,3#^, Chunxu Chen^1,2^, Stephan William Anderson^2,3^, Xin Zhang^1,2^*

^1^Department of Mechanical Engineering, Boston University, Boston, MA 02215, USA.

^2^Photonics Center, Boston University, Boston, MA 02215, USA.

^3^Department of Radiology, Boston University Medical Campus, Boston, MA 02118, USA.

^#^These authors contributed equally.

*Corresponding author:

Xin Zhang

110 Cummington Mall, Boston, MA 02215, USA

Phone: +1(617) 358-2702

Fax: +1(617) 353-5866

E-mail: xinz@bu.edu

E-mail addresses of all authors:

Ke Wu: wk0305ok@bu.edu

Guangwu Duan: duangw@bu.edu

Xiaoguang Zhao: zhaoxg@bu.edu

Chunxu Chen: chunxc@bu.edu

Stephan William Anderson: sande@bu.edu

**Resistance derivation for the helical coil**

In order to evaluate the degree of field enhancement ratio and power transfer efficiency improvement by the metamaterial or M-LFEP, it is necessary to model the helical coil featured in both the unit cell of the metamaterial and the local resonator with an RLC circuit. In the case of a helical coil, the power consumed by the coil is not only dissipated internally but also radiates externally. As a result, there are absorptive and radiative resistances, and their standard formulas are presented below:

$$\begin{aligned} R_{0}=\frac{l_{t}}{2a}\sqrt{\frac{\rho f\mu_{0}}{\pi}}+\frac{2\varepsilon_{i}}{\omega C_{eff}\left( \varepsilon_{r}+1 \right)^{2}}\#\left( S1 \right) \end{aligned}$$

$$\begin{aligned} l_{t}=\sqrt{\left( n2\pi r \right)^{2}+h^{2}}\#\left( S2 \right) \end{aligned}$$

$$\begin{aligned} R_{rad}=\sqrt{\frac{\mu_{0}}{\varepsilon_{0}}}\left[ \frac{8n^{2}\pi^{5}}{3}\left( \frac{r}{\lambda} \right)^{4}+\frac{4}{3\pi}\left( \frac{h}{\lambda} \right)^{2} \right]\#\left( S3 \right) \end{aligned}$$

in which, *l_t_* is the total length of the electrically conducting wire, *a* is the radius of conducting wire, *h* is the height of helix, *r* is the radius of helix, *ρ* is the resistivity of conducting wire, *f* is frequency, *μ_0_* is the permeability in free space, *ε_0_* is the permittivity in free space, *λ* is the wavelength, *C_eff_* is the equivalent capacitance of the helix, and *ε_r_* and *ε_i_* represent the real and imaginary permittivities of the scaffolding material, respectively. The ohmic resistance of the unit cells in Eq. S1 includes both the ohmic loss from the conducting wire as well as the dielectric loss from the scaffolding core. The first term in Eq. S3 represents a magnetic dipole radiation term and the second term is due to the electronic dipole of the helix.

**Magnetic field enhancement ratio by metamaterial or M-LFEP**

In order to calculate the magnetic field enhancement ratio due to the metamaterial or M-LFEP, Faraday's law will be employed to calculate the induced voltage along the helical coil. Following derivation of the induced voltage, the induced current may be derived using Ohm's law. Subsequently, the magnetic field generated by the induced current may be calculated using the Biot–Savart law. Next, the field enhancement ratio may be derived by adding the induced magnetic field generated by the metamaterial or the M-LFEP and the initial field *H_0_* generated by feeding loop together, followed by normalizing the sum by the initial magnetic field *H_0_*.

Firstly, the induced voltage along the helical coil may be calculated as follows:

$$\begin{aligned} V=-j\omega n\pi r^{2}\mu_{0}H_{0}\#\left( S4 \right) \end{aligned}$$

where *ω* is angular frequency, *n* is the number of turns of helical coil, and *r* is the radius of the helix. Furthermore, the induced current along the helix may be calculated as follows:

$$\begin{aligned} I=\frac{V}{j\omega L_{eff}+\frac{1}{j\omega C_{eff}}+R_{o}+R_{rad}}\#\left( S5 \right) \end{aligned}$$

$$\begin{aligned} L_{eff}=L_{s}+\kappa\sum L_{m}\#\left( S6 \right) \end{aligned}$$

$$\begin{aligned} C_{eff}=C_{s}+\kappa\sum C_{m}\#\left( S7 \right) \end{aligned}$$

where *L_eff_* and *C_eff_* represent the equivalent inductance and capacitance of the helical coil (including the self-reactance denoted with subscript *s* and the mutual reactance denoted with the subscript *m* ), respectively. *κ* indicates the discrepancy of the induced current in different unit cells of the metamaterial. By observing the equation Eq. S5, when *ω^2^=1/(L_eff_×C_eff_*), the induced current in the helix reaches its maximum value and the coil array functions in a resonant state. At resonance, the current exhibits a sinusoidal profile along the length of the conducting wire, with a maximum value at the middle and a value of zero at the two ends. Based on this analysis, the current density along the helical coil can be calculated by the amplitude of the current (*I0*) in the middle of the helix and the parameterization coordinate along the length of the conducting wire. For the amplitude of current (*I0*), the maximum value is $\sqrt{2}$-fold of the mean spatial current value, which can be calculated with Eq. S5. To simplify the calculation process, the helix is placed in cylindrical coordinates, with the helix along the z-axis from –*h*/2 to *h*/2, as shown in Fig. S1. In this case, the current density at position *z’* may be expressed as:

$$\begin{aligned} I_{d}=cos\left( \frac{\pi z^{'}}{h} \right)\frac{nI_{0}}{h}\#\left( S8 \right) \end{aligned}$$

After derivation of the current in the metamaterial, the corresponding induced magnetic field at any given point in space may be calculated using the Biot-Savart law, which may be expressed as:

$$\begin{aligned} \vec{B}=\frac{\mu_{0}nI_{0}}{4\pi h}\int_{-h/2}^{h/2} \cos\left( \frac{\pi z^{'}}{h} \right)dz^{'}\oint\frac{dx^{'}\times\vec{R}}{\left| \vec{R} \right|^{3}}\#\left( S9 \right) \end{aligned}$$

in which $\vec{R}$ is the distance between the target point and the integral elements in the vector. Assuming the coordinate of the target point in free space is (*ρ, φ, z*) in polar coordinates, the magnetic field equation for this point may be expanded into two components, which express the magnetic field in axial and radial directions, respectively, as follows:

$$\begin{aligned} B\left( z \right)=\frac{\mu_{0}nI_{0}}{4\pi h}\int_{-h/2}^{h/2} \cos\left( \frac{\pi z^{'}}{h} \right)\int_{0}^{2\pi} \frac{\left( r-\rho cos\varphi^{'} \right)}{\left[ \rho^{2}-2r\rho cos\varphi^{'}+r^{2}+\left( z-z^{'} \right)^{2} \right]^{3/2}}d\varphi^{'}dz^{'}\#\left( S10 \right) \end{aligned}$$

$$\begin{aligned} B\left( \rho\right)=\frac{\mu_{0}nI_{0}}{4\pi h}\int_{-h/2}^{h/2} \cos\left( \frac{\pi z^{'}}{h} \right)\int_{0}^{2\pi} \frac{\left( z-z^{'} \right)cos\varphi^{'}}{\left[ \rho^{2}-2r\rho cos\varphi^{'}+r^{2}+\left( z-z^{'} \right)^{2} \right]^{3/2}}d\varphi^{'}dz^{'}\#\left( S11 \right) \end{aligned}$$

Since the sensor antenna was aligned to the axial direction of magnetic field, we only consider the field enhancement ratio along the axial direction. In terms of the enhancement ratio from the metamaterial, the magnetic field generated by all the unit cells in metamaterial need to be taken into consideration. As a result, the field enhancement ratio for the metamaterial may be expressed by:

$$\begin{aligned} \nu_{mm}=\frac{\sum\frac{a_{i}}{a_{1}}B\left( z \right)+B_{0}}{B_{0}}\#\left( S12 \right) \end{aligned}$$

in which *a_i_* represent the resonant strength of each unit cell in the metamaterial, which may be derived by Eq.4 and *a_4_* is the resonant strength of the unit cell located in the center of metamaterial. The results of the aforementioned calculation are plotted in Fig. 4b. With regards to the field enhancement ratio due to the M-LFEP, this may be expressed by Eq. S13, as follows, with the calculation results plotted in Fig. 5d.

$$\begin{aligned} \nu_{lr}=\frac{B\left( z \right)+B_{0}}{B_{0}}\#\left( S13 \right) \end{aligned}$$

All of the parameters employed in the derivation process in the case of the unit cell of the metamaterial are listed in Table S1, while those employed in the derivation process for the M-LFEP are listed in Table S2.

Fig. S1. Schematic drawing of a helix representing a unit cell of the metamaterial, labeled with parameters used in Eq. S8 to S13 to derive the magnetic field strength.

Table S1. Parameters employed in resistance derivation for unit cell of metamaterial.

| *a* (mm) | 0.125 | *ε_r_* | 1.46 | *n* | 3.85 |
| --- | --- | --- | --- | --- | --- |
| *h* (mm) | 5.39 | *ε_i_* | 0.05 | *Ceff* (F) | 3.24×10^-13^ |
| *r* (mm) | 4.5 | *ρ* (Ω*m) | 1.68×10^-8^ | *Leff* (H) | 9.35×10^-8^ |
| *μ_0_* (N/A^2^) | 4π×10^-7^ | *ε_0_* (F/m) | 8.854×10^-12^ |  |  |

Table S2. Parameters employed in resistance derivation for M-LFEP.

| *a* (mm) | 0.01 | *ε_r_* | 1.3 | *n* | 7 |
| --- | --- | --- | --- | --- | --- |
| *h* (mm) | 3.5 | *ε_i_* | 0.07 | *Ceff* (F) | 1.79×10^-13^ |
| *r* (mm) | 2.5 | *ρ* (Ω*m) | 1.59×10^-8^ | *Leff* (H) | 1.69×10^-7^ |

**Modeling of the integrated chip**

In this work, the micro integrated chip serves as a load in the analysis of the power transfer efficiency, and as a verification of the RFID communication. An off-the-shelf RFID IC chip (SL3ICS1002/1202, NXP semiconductors Inc.) integrated with a miniaturized double layer spiral antenna is used as a communication module for the integrated tag, shown in Fig. S2a. In order to optimize the resonance frequency of the integrated tag close to 915MHz, the integrated tag is modeled with an equivalent RLC circuit. In this circuit, *R_l_* and *C_l_* are the effective resistance and capacitance of RFID chip, respectively. Based on its datasheet, the RFID chip’s values of *R_l_* and *C_l_* are 18.6Ω and 1.0186pF, respectively. An inductance *L_a_* serially connected with a resistance *R_a_* is employed to model the spiral antenna, and *V_a_* represents the induced electromotive force (emf) in the sensor antenna when placed in an alternating magnetic field as shown in Fig. S2b. The value of the resistance *R_a_* may be expressed as:

$$\begin{aligned} R_{a}=\frac{2\rho_{a}l_{a}}{2\left( w+t \right)\cdot\left( \frac{\kappa\delta}{2} \right)}\#\left( S14 \right) \end{aligned}$$

$$\begin{aligned} l_{a}=2n_{a}\left( d_{out}+d_{in} \right)\#\left( S15 \right) \end{aligned}$$

$$\begin{aligned} d_{in}=d_{out}-2n_{a}w-2\left( n_{a}-1 \right)s\#\left( S16 \right) \end{aligned}$$

$$\begin{aligned} \delta=\sqrt{\frac{\rho}{\left( \pi f\mu_{0} \right)}}\#\left( S17 \right) \end{aligned}$$

in which *ρ_a_* is the resistivity of copper, *w* is the width of the copper wire, and *t* is thickness of copper wire, which are illustrated in Fig. S2c. κ indicates the degree of concentration of the surface current, *l_a_* is the total length of copper wire in each layer expressed with Eq. S15, in which *n_a_* is the number of turns of each layer, *d_out_* is the outer side length of the square spiral antenna, *d_in_* is the inner sider length expressed by Eq. S16, and *s* is the space between two adjacent turns. *δ* is the skin depth expressed in Eq. S17, in which *f* is the working frequency, ρ is the resistivity of copper, and μ_0_ is the permeability in vacuum. The effective inductance *L_a_* of the sensor antenna may be expressed by:

$$\begin{aligned} L_{a}=L_{top}+L_{bottom}+2k_{c}\sqrt{L_{top}L_{bottom}}\#\left( S18 \right) \end{aligned}$$

$$\begin{aligned} L_{top}=L_{bottom}=\frac{\mu_{0}{n_{a}}^{2}d_{aver}c_{1}}{2}\left[ ln\left( \frac{c_{2}}{p} \right)+c_{3}p+c_{4}p^{2} \right]\#\left( S19 \right) \end{aligned}$$

$$\begin{aligned} p=\frac{\left( d_{out}-d_{in} \right)}{\left( d_{out}+d_{in} \right)}\#\left( S20 \right) \end{aligned}$$

in which *L_top_* and *L_bottom_* are the effective inductances of the top and bottom layer, respectively, in this dual-layer spiral antenna. *k_c_* denotes the coupling coefficient, which can be assumed to be 1 due to the negligibly small distance between the two layers of the antenna with respect to their side length. *c*_1_ to *c*_4_ are constants related to the antenna configuration in Table S3. The magnetically-induced voltage across the sensor antenna may be expressed as:

$$\begin{aligned} V_{a}=-j2\omega B_{z}S\cdot\cos\left( \varphi\right)=-j2\omega\frac{\mu_{0}b^{2}I_{0}}{\pi\left( b^{2}+z^{2} \right)^{3/2}}S\cdot\cos\left( \varphi\right)\#\left( S21 \right) \end{aligned}$$

$$\begin{aligned} S=\sum_{i=1}^{n_{a}} \left[ d_{out}-2\left( w+s \right)\left( i-1 \right) \right]^{2} \\ =n_{a}{d_{out}}^{2}-2\left( w+s \right)d_{out}n_{a}\left( n_{a}-1 \right)+4\left( w+s \right)^{2}\frac{n_{a}\left( n_{a}-1 \right)\left( 2n_{a}-1 \right)}{6}\#\left( S22 \right) \end{aligned}$$

in which *φ* is the misalignment angle between the orientation of the sensor antenna and magnetic field in the axial direction; here, *φ* is 0. *S* is the cross-sectional area of the single layer in the spiral sensor antenna. The power received by RFID tag may be expressed by:

$$\begin{aligned} P_{L}=\frac{1}{2}\left| \frac{V_{a}}{R_{a}+R_{L}+j\omega L_{a}+\frac{1}{\left( j\omega C_{L} \right)}} \right|^{2}\cdot R_{L}\#\left( S23 \right) \end{aligned}$$

By substituting Eq. R1 – R9 and all the numbers in Table S3 into Eq. S23, the relationship between the geometry of the spiral antenna (number of turns and outer side length of the spiral) and power received by the RFID chip at the working frequency of 915 MHz is derived. The result is normalized to its maximum value and plotted in **Fig. S2d**. For every specific outer side length of the spiral, there is a peak of received power achieved at a certain number of turns, at which the imaginary part of sensor antenna cancels that of the RFID chip, and the peak value increases as the side length of the spiral increases. Next, the theoretical conclusions are validated with simulations in CST Microwave Studio, as shown in **Fig. S2e**. A high degree of agreement between the analytical model and simulation results can be observed, proving the validity of the theoretical model. To control the antenna size within 0.5 mm, we chose the optimized point with 4 turns and 0.355 mm outer side length as our design reference. Lastly, the miniaturized spiral antenna is fabricated using multiple photolithography and copper electroplating steps. The fabrication result are depicted in Fig. S2f. All parameters employed in the derivation process for the spiral antenna are listed in Table S3.

**Modeling of the feeding loop**

The loop antenna was connected to a control circuit through a 50ohm coaxial transmission line and an AC current was fed to the loop antenna to generate an alternating magnetic field in order to evaluate the system power transfer efficiency. The effective resistance may be expressed by:

$$\begin{aligned} R_{fl}=\frac{b}{a_{loop}}\sqrt{\pi f\rho\mu_{0}}+\sqrt{\frac{\mu_{0}}{\varepsilon_{0}}}\frac{8\pi^{5}}{3}\left( \frac{b}{\lambda} \right)^{4}\#\left( S24 \right) \end{aligned}$$

in which *b* is the radius of the loop antenna with value of 25mm, *a_loop_* is the radius of copper wire with value of 0.51mm. The first term in Eq. S24 represents the ohmic loss and the second term represents the radiation loss of the loop antenna.

Table S3. Parameters employed in modeling of sensor antenna.

| *w* (μm) | 10 | $c_{1}$ | 1.27 | *k_s_* | 1 |
| --- | --- | --- | --- | --- | --- |
| *s* (μm) | 10 | $c_{2}$ | 2.07 | *ρ* (Ω*m) | 1.68×10^-8^ |
| *t* (μm) | 5 | $c_{3}$ | 0.18 | κ | 1.17 |
| *n_a_* | 3.875 | $c_{4}$ | 0.13 | *d_out_* (μm) | 380 |


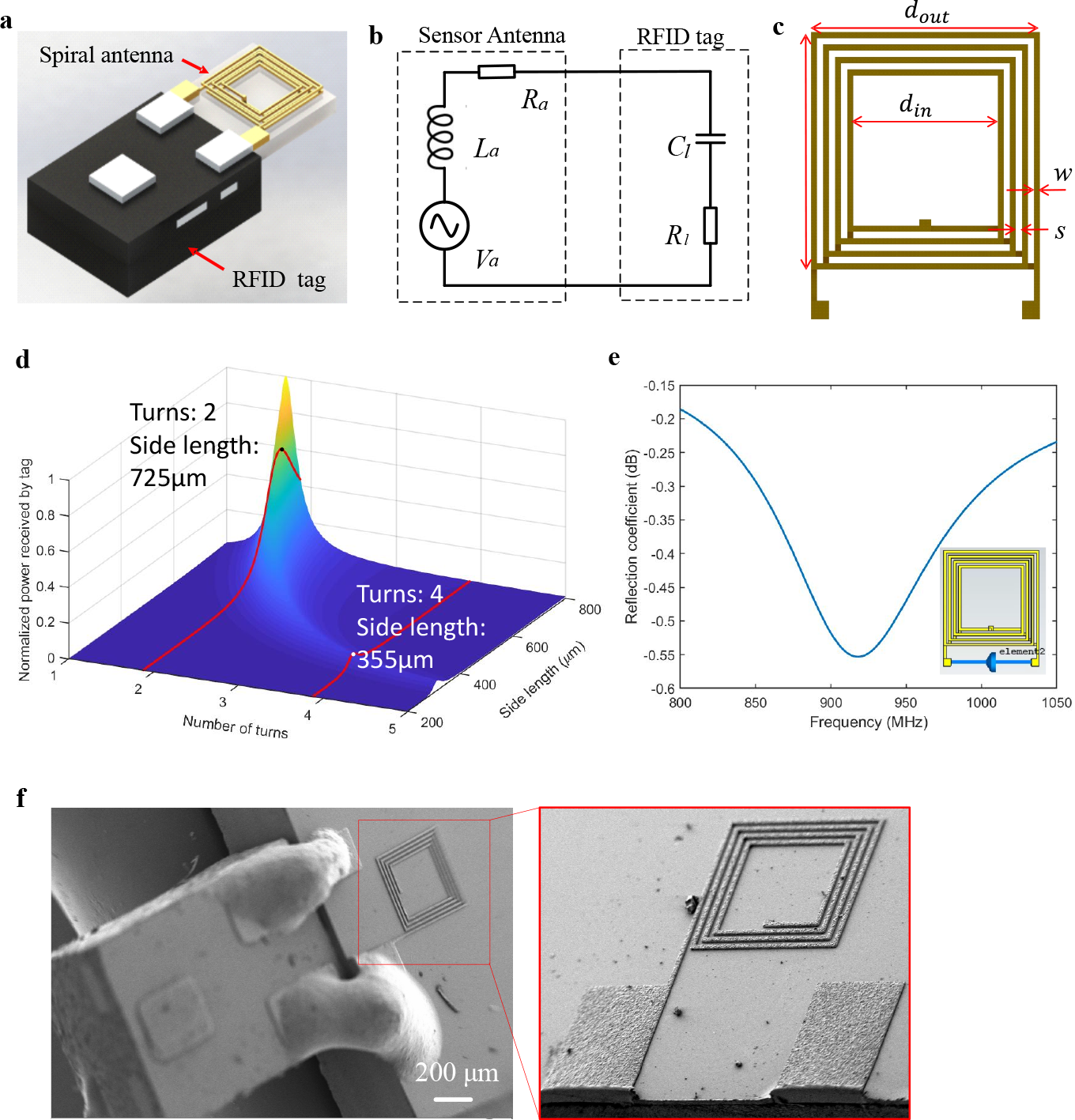


Fig. S2. **a**, Conceptual drawing of the integrated chip. **b**, Lumped circuit model of the integrated chip. **c**, Sensor antenna labeled with parameters used in Eq. R1 to R9. **d**, Normalized value of power received by RFID chip versus number of turns and side length of the miniaturized antenna. **e**. Simulation results to analyze the resonance frequency of the integrated tag. The inset depicts the simulation model of the sensor antenna with 4 turns. **f**, SEM image of a fabricated integrated tag, featuring micro tag integrated with sensor antenna. Inset shows a detailed SEM image of the fabricated double layer spiral antenna.

**Power transfer efficiency derivation**

The ultimate goal of this work was to enhance the magnetic field and thereby improve power transfer efficiency using a metamaterial or M-LFEP. In order to explicitly and rigorously formulate the concept of power transfer efficiency enhancement by the metamaterial or M-LFEP, it is necessary to derive the expressions of system efficiency. As stated in the paper, we theoretically designed three setups for the readout platform to investigate the enhancement effects using the metamaterial or local resonator separately. The first setup, employed as a reference, featured a loop antenna as the reader antenna and a double layer spiral antenna as sensor antenna, without the use of either the metamaterial or M-LFEP. For the second setup, the metamaterial was placed in the center of loop antenna to form a metamaterial-enhanced reader antenna, and the same double layer spiral antenna was employed as the sensor antenna without the use of the M-LFEP. The third set up employed the loop antenna as the reader antenna and a double layer spiral antenna as the sensor antenna, however, the M-LFEP was added to improve power transfer efficiency. To simplify the calculation process, the reader antenna was placed in cylindrical coordinates (*r, θ, z*), as shown in Fig. S3. An alternating current *I_0*_sinωt* was fed into the loop antenna to provide an alternating magnetic field with a frequency of 915MHz for this power transfer system. As the frequency of feeding the current was 915MHz, the current inside the feeding loop may be considered as a standing wave with sinusoidal distribution, whose amplitude along the copper wire may be expressed as:

$\begin{aligned} I\left( \theta\right)=I_{0}\cdot sin\left( \frac{\theta}{2} \right)\#\left( S25 \right) \end{aligned}$For the first setup, the magnetic field strength at any point generated by a current element at (*b*, *θ, 0*) in the loop antenna may be expressed as:

$$\begin{aligned} \vec{dB}=\frac{\mu_{0}I_{0}\sin\left( \frac{\theta}{2} \right)}{4\pi}\cdot\left( \frac{\vec{dl}\times\vec{R}}{R^{3}} \right)\#\left( S26 \right) \end{aligned}$$

in which $\vec{R}$ is the distance between the target point and the integral elements in the vector. When investigating the impact of distance between the reader and sensor antennas on power transfer efficiency, we only considered the condition in which the sensor antenna moved along the z-axis. Thus, the magnetic field strength on the z-axis generated by feeding loop may be expressed as:

$$\begin{aligned} \vec{B}=\oint\vec{dB}=\int_{0}^{2\pi} \left[ \frac{\mu_{0}I_{0}\sin\left( \frac{\theta}{2} \right)bz\cdot\vec{a_{r}}}{4\pi\left( b^{2}+z^{2} \right)^{\frac{3}{2}}}+\frac{\mu_{0}I_{0}\sin\left( \frac{\theta}{2} \right)b^{2}\cdot\vec{a_{z}}}{4\pi\left( b^{2}+z^{2} \right)^{\frac{3}{2}}} \right]d\theta\#\left( S27 \right) \end{aligned}$$

The first term in Eq. S27 is the magnetic field strength in the radial $\vec{r}$ direction and the second term is the magnetic field strength in the axial $\vec{z}$ direction. In order to further simplify the calculation process, we assumed that the spiral antenna was placed horizontally such that the induced voltage in the sensor antenna could only arise from the magnetic field components along axial direction, which can be expressed as:

$$\begin{aligned} B_{z}=\int_{0}^{2\pi} \frac{\mu_{0}I_{0}\sin\left( \frac{\theta}{2} \right)b^{2}\cdot\vec{a_{z}}}{4\pi\left( b^{2}+z^{2} \right)^{3/2}}d\theta=\frac{\mu_{0}b^{2}I_{0}}{\pi\left( b^{2}+z^{2} \right)^{3/2}}\#\left( S28 \right) \end{aligned}$$

The system efficiency for the first setup may be expressed by:

$$\begin{aligned} \eta_{1}=\frac{P_{L1}}{P_{L1}+P_{loss_{fl}}+P_{loss_{a1}}}\#\left( S29 \right) \end{aligned}$$

$$\begin{aligned} P_{L1}=\frac{1}{2}\left| \frac{V_{a}}{R_{a}+R_{L}+j\omega L_{a}+\frac{1}{\left( j\omega C_{L} \right)}} \right|^{2}\cdot R_{L}\#\left( S30 \right) \end{aligned}$$

$$\begin{aligned} P_{loss\_a1}=\frac{1}{2}\left| \frac{V_{a}}{R_{a}+R_{L}+j\omega L_{a}+\frac{1}{\left( j\omega C_{L} \right)}} \right|^{2}\cdot R_{a}\#\left( S31 \right) \end{aligned}$$

$$\begin{aligned} P_{loss\_fl}=\frac{1}{2}{I_{0}}^{2}\left( R_{fl_{ohm}}+R_{r_{radiation}} \right)\#\left( S32 \right) \end{aligned}$$

in which *P_L1_*, *P_loss_a1_*, and *P_loss_fl_* are the power received by RFID tag, the power dissipated in sensor antenna, and the power loss in the loop antenna, respectively. The calculation results are presented in Fig. 6(a).

Regarding the second setup, the metamaterial was placed in the center of the loop antenna to enhance the initial magnetic field. When evaluating the power transfer efficiency, the power loss in the metamaterial needs to be taken into consideration. The power loss in helical coils of the array of the metamaterial may be expressed by:

$$\begin{aligned} P_{loss\_mm}=\frac{1}{2}\sum_{i=1}^{7} \left[ {I_{mm_{i}}}^{2}\left( R_{o\_mm}+R_{rad\_mm} \right) \right]=\frac{1}{2}\sum_{i=1}^{7} \left[ \left( \frac{a_{i}}{a_{1}}I_{mm_{1}} \right)^{2}\cdot\left( R_{o\_mm}+R_{rad\_mm} \right) \right]\#\left( S33 \right) \end{aligned}$$

in which *R_o_mm_* and *R_rad_mm_* are the absorption and radiation resistance of the unit cells in metamaterial, respectively, which may be calculated using Eq.S1 and Eq.S3. The induced current *I_mm_1_* in the unit cell located in the center of the metamaterial may be expressed by:

$$\begin{aligned} I_{mm_{1}}=\frac{-j\omega n_{mm}\pi{r_{mm}}^{2}\cdot\left. B_{z} \right|_{\left( z=0 \right)}}{R_{o\_mm}+R_{rad\_mm}+j\omega L_{cell}+\frac{1}{\left( j\omega C_{cell} \right)}}\#\left( S34 \right) \end{aligned}$$

The power received by the RFID tag following enhancement by the metamaterial may be expressed as modifications of Eq. S29 and Eq. S30 as follows:

$$\begin{aligned} P_{L2}=\frac{1}{2}{\nu_{mm}}^{2}\left( f,z \right)\left| \frac{V_{a}}{R_{a}+R_{L}+j\omega L_{a}+\frac{1}{\left( j\omega C_{L} \right)}} \right|^{2}\cdot R_{L}\#\left( S35 \right) \end{aligned}$$

$$\begin{aligned} P_{loss\_a2}=\frac{1}{2}{\nu_{mm}}^{2}\left( f,z \right)\left| \frac{V_{a}}{R_{a}+R_{L}+j\omega L_{a}+\frac{1}{\left( j\omega C_{L} \right)}} \right|^{2}\cdot R_{a}\#\left( S36 \right) \end{aligned}$$

in which *ν_mm_(f, z)* is the magnetic field enhancement ratio due to metamaterial. The enhancement ratio is a function of the working frequency *f* and separation distance *z* between the reader and sensor antennas, which can be derived using Eq. S12. As a result, the power transfer efficiency of the second setup may be expressed as follows:

$$\begin{aligned} \eta_{2}=\frac{P_{L2}}{P_{L2}+P_{loss\_fl}+P_{loss\_a2}+P_{loss\_mm}}\#\left( S37 \right) \end{aligned}$$

The calculation results are plotted in Fig. 6(b).

For the third setup, the M-LFEP was employed to boost the magnetic field in the vicinity of the sensor antenna. The power loss in the M-LFEP is not trivial when evaluating the system power transfer efficiency and may be expressed as:

$$\begin{aligned} P_{loss\_lr}=\frac{1}{2}{I_{lr}}^{2}\left( R_{o\_lr}+R_{rad\_lr} \right)\#\left( S38 \right) \end{aligned}$$

in which *Ro_lr, Rrad_lr* are the absorption and radiation resistance of the M-LFEP, respectively, which maybe calculated by Eq.S1 and Eq.S3. *I_lr* is the induced current in M-LFEP, which may be expressed by:

$$\begin{aligned} I_{lr}=\frac{V_{lr}}{R_{o_{lr}}+R_{rad_{lr}}+j\omega L_{lr}+\frac{1}{\left( j\omega C_{lr} \right)}}=\frac{-j\omega n_{lr}\pi{r_{lr}}^{2}B_{z}}{R_{o\_lr}+R_{rad\_lr}+j\omega L_{lr}+\frac{1}{\left( j\omega C_{lr} \right)}}\#\left( S39 \right) \end{aligned}$$

Similarly, the power received by the RFID tag with the enhancement due to the presence of the M-LFEP may be expressed as modifications of Eq. S30 and Eq. S31 as follows:

$$\begin{aligned} P_{L3}=\frac{1}{2}{\nu_{lr}}^{2}\left( f \right)\left| \frac{V_{a}}{R_{a}+R_{L}+j\omega L_{a}+\frac{1}{\left( j\omega C_{L} \right)}} \right|^{2}\cdot R_{L}\#\left( S40 \right) \end{aligned}$$

$$\begin{aligned} P_{loss\_a3}=\frac{1}{2}{\nu_{lr}}^{2}\left( f \right)\left| \frac{V_{a}}{R_{a}+R_{L}+j\omega L_{a}+\frac{1}{\left( j\omega C_{L} \right)}} \right|^{2}\cdot R_{a}\#\left( S41 \right) \end{aligned}$$

in which *ν_lr_(f)* is the magnetic field enhancement ratio due to the M-LFEP. Since the sensor antenna and the M-LFEP, fixed as a unit, are integrated together, the value of the ratio will not change as a function of separation distance, and may be derived using Eq. S13. The power transfer efficiency of the third setup may be expressed by:

$$\begin{aligned} \eta_{3}=\frac{P_{L3}}{P_{L3}+P_{loss\_fl}+P_{loss\_a3}+P_{loss\_lr}}\#\left( S42 \right) \end{aligned}$$

The calculation results are presented in Fig. 6(c).

**Comparison with prior reported work in near-field UHF RFID**

We developed an efficient UHF RFID readout platform enhanced by metamaterial for the interrogation of passive microsensor tags. Benefitting from the magnetic field enhancement approaches of our readout platform, it is feasible to radically miniaturize the RFID tag and tag antenna. While the performance of near-field-based RFID systems are difficult to compare due to differences in coupling distances, operation frequencies, reader antenna configurations and power dissipation of the RFID tags, we nevertheless offer a summary of the state-of-the-art in UHF RFID technology with systems featuring miniaturized tag antennas. Table S4 demonstrates an analysis of miniaturized UHF RFID tags in terms of tag antenna size.

Table S4. Analysis of state-of-the-art miniaturized passive UHF RFID tag antennas.

| Ref. | Antenna size (mm^2^) | Working frequency (MHZ) | Read range (mm) | RF power of reader |
| --- | --- | --- | --- | --- |
| [S1] | 4×4 | 915 | 13mm | 20dBm |
| [S2] | 1×1 | 900 | 10mm | 30dBm |
| [S3] | 0.6×0.6 | 900 | 2mm | 20dBm |
| [S4] | 0.55×0.55 | 900 | 4mm | 225mA in reader antenna |
| This work | 0.38×0.38 | 915 | 3.7mm | 20dBm |

Fig. S3. Schematic drawing of the reader and sensor antennas labeled with parameters used in Eq. S22 to S41 to derive the system power transfer efficiency.


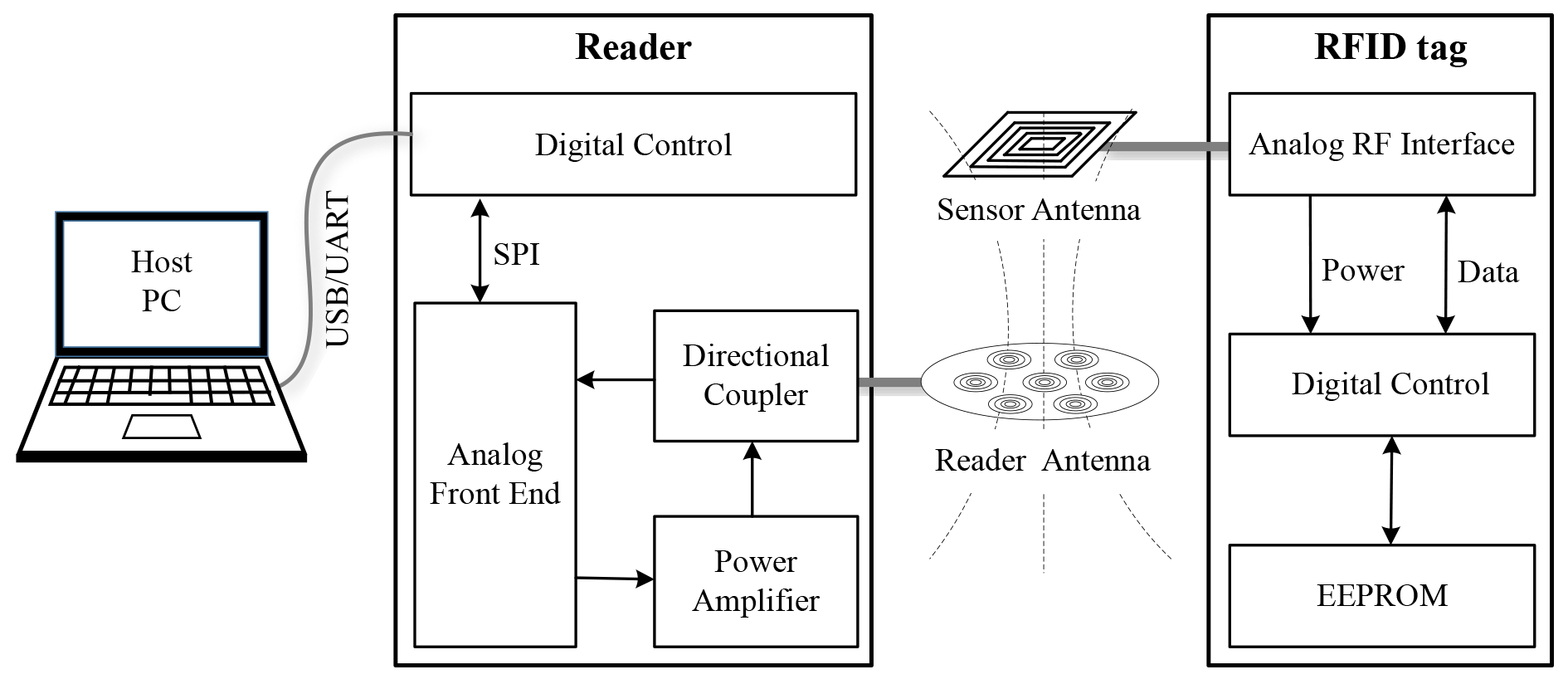


Fig. S4. The block diagram of the RFID system.

**RFID readout process**

The simplified architecture of the RFID system comprises two principal modules: the reader unit and the tag unit. The basic block diagram of the RFID reader and tag modules is shown in Fig. R6. The reader module circuit has two main parts: the RF transceiver unit and the control unit. The main functions of the control unit are data processing and management, communication with the host server and tags, and execution of the commands from the server software. The RF transceiver unit has a power amplifier (PA), a coupler for isolation, and an RFID reader IC (AS3993), which integrates transmission, reception, and protocol processing of RFID. Once this reader IC receives the commands from the host server, it will generate a radio frequency (RF) signal; the RF signal could be amplified by the PA and then fed to the reader antenna. Through magneto-resonance coupling between the reader and sensor antennas, the RF signal can be coupled to the sensor antenna. On the RFID tag side, the tag (SL3ICS1002/1202 IC) consists of three major blocks: analog RF interface, digital controller, and EEPROM. The RF signal received by the analog part in the tag could be rectified and demodulated as power and data, which provides stable supply voltage and received information for subsequent processing by the digital components. The digital section includes the state machines, processes the protocol and handles communication with the EEPROM, which contains the communication protocol and the user data. Furthermore, the modulation transistor in the analog portion serves to transmit data back to the reader. The backscattering signal is collected by the reader antenna and then demodulated by a demodulator in the reader module to recover the data sent by tag. Finally, the control unit in the reader module will process these data and communicate with the host server, which displays the data sent by the tags.

**Reference**

[S1] He, Y., Zhao, X., Zhang, C., & Wang, Z. A fully integrated chip-ID tag used in chip information identification. *Proc. IEEE Int. Conf. Radio Frequency Identification*, 172–176 (2012).

[S2] Xi, J. et al., On-chip antenna design for UHF RFID. *Electron. Lett.* **45**, 14-16 (2009).

[S3] Peng, Q. et al. A low cost UHF RFID system with OCA tag for short range communication. *IEEE Trans. Ind. Electron.* **62**, 4455–4465 (2015).

[S4] Shameli, A. et al. A UHF near-field RFID system with fully integrated transponder. *IEEE Trans. Microw. Theory Tech.* **56**, 1267-1277 (2008).
